# Mechanisms of assembly and genome packaging in an RNA virus revealed by high-resolution cryo-EM

**DOI:** 10.1038/ncomms10113

**Published:** 2015-12-10

**Authors:** Emma L. Hesketh, Yulia Meshcheriakova, Kyle C. Dent, Pooja Saxena, Rebecca F. Thompson, Joseph J. Cockburn, George P. Lomonossoff, Neil A. Ranson

**Affiliations:** 1 Astbury Centre for Structural Molecular Biology, University of Leeds, Mount Preston Street, Leeds LS2 9JT, UK; 2 Department of Biological Chemistry, John Innes Centre, Norwich Research Park, Colney, Norwich NR4 7UH, UK

## Abstract

Cowpea mosaic virus is a plant-infecting member of the *Picornavirales* and is of major interest in the development of biotechnology applications. Despite the availability of >100 crystal structures of *Picornavirales* capsids, relatively little is known about the mechanisms of capsid assembly and genome encapsidation. Here we have determined cryo-electron microscopy reconstructions for the wild-type virus and an empty virus-like particle, to 3.4 Å and 3.0 Å resolution, respectively, and built *de novo* atomic models of their capsids. These new structures reveal the C-terminal region of the small coat protein subunit, which is essential for virus assembly and which was missing from previously determined crystal structures, as well as residues that bind to the viral genome. These observations allow us to develop a new model for genome encapsidation and capsid assembly.

## Abstract (web summary)

Little is known about how the plant-infecting cowpea mosaic virus (CPMV)—an invaluable tool in several biotechnology applications—packages its single-strand RNA genome into the capsid. Here the authors present two high-resolution cryo-EM structures of CPMV and a new model for RNA recognition and capsid assembly.

## Graphical abstract

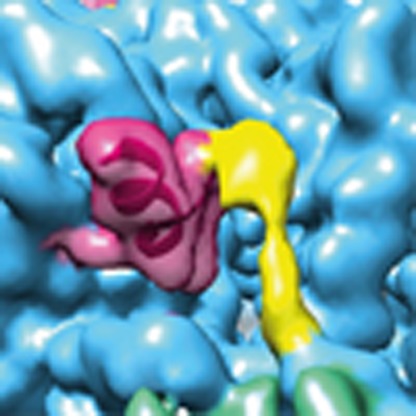

A crucial step in virus assembly is the specific encapsidation of the genome. This is a particular challenge for single-stranded RNA viruses, as they must preferentially select their genomes from a high background of cellular mRNA. Owing to their importance as pathogens and their relatively simple, non-enveloped capsids, members of the order *Picornavirales* have been extensively studied. These viruses include members infecting vertebrates (poliovirus, hepatitis A virus), insects (deformed wing virus) and plants (cowpea mosaic virus (CPMV)) and >100 X-ray structures of their capsids are available^1^. However, while these reveal the structure of the coat proteins in exquisite detail, they give very few clues about how RNA is packaged or how specificity is achieved. Despite many years of study on the replication cycle of the *Picornavirales*, remarkably little is known about the process of RNA encapsidation. For example, it is not known which type of coat protein aggregate is required for efficient RNA incorporation or what controls the specificity of RNA packaging^2^.

CPMV, the type member of the *Comoviridae* subfamily of the plant-infecting *Secoviridae*, has a bipartite, positive-sense, single-stranded RNA genome. The two segments, RNA-1 (6 kb) and RNA-2 (3.5 kb) (Fig. 1a), are separately encapsidated. Its icosahedral particles have a maximum diameter of ∼30 nm and are comprised of 60 copies each of a Large (L) and Small (S) coat protein (Fig. 1). L and S are processed from a single precursor polyprotein, the RNA-2-encoded VP60, by the action of the RNA-1-encoded 24K proteinase. Particles containing the two different genomic RNAs can readily be purified (Fig. 1c). Crystallographic structures are available for three comoviruses^3^: CPMV^4^,^5^, bean pod mottle virus (BPMV)^6^ and red clover mottle virus^7^. Together, the L and S subunits comprise three β-barrel domains (two from L, one from S; Fig. 1d) corresponding to the three quasi-equivalent conformers of a *T*=3 icosahedral lattice. Comovirus capsids thus adopt a p*T*=3 quasi symmetry (Fig. 1e), forming a particle with pronounced turrets formed from the S subunit at the particle fivefold axes. Indeed, a penton of L and S subunits appears to be the basic building block for all *Picornavirales* capsids^2^. While the capsid structure is well-understood, the organization of encapsidated genomic RNA is not. In only one instance, the RNA-2-containing component of BPMV, can details of RNA structure be observed^6^, where density for 11 ordered ribonucleotides near the particle threefold axes was visualized^6^. This RNA forms a trefoil, and binds in a complementary depression on the inside of the capsid. Owing to icosahedral averaging it was impossible to deduce the RNA sequence, but the base composition was not random, and it was suggested that these sequences might be critical determinants for assembly or stability of capsids^8^.

The mechanisms by which RNA is selected and packaged are also poorly understood. The only portion of the CPMV capsid proteins currently implicated in RNA packaging is a segment of 24 amino acids at the C terminus of the S subunit^9^. This sequence is proteolytically cleaved during maturation without affecting particle stability or infectivity^10^, and is therefore missing from X-ray structures of CPMV^4^. The suggestion that this region may promote interaction with RNA was supported by the fact that the S subunit loses its suppressor of host RNA silencing activity when the C terminus is removed^11^. However, from studies using a system that can produce RNA-free CPMV capsids in the absence of infection^12^ it is clear that this extension is also essential for capsid assembly itself^13^. Deletion of the entire 24-amino acid sequence almost completely abolished empty particle formation. This effect could be partially reversed by substituting the 24 amino acids with 6 histidines, suggesting that basic amino acids in the C-terminal extension may play a role in capsid assembly^13^.

In recent years, the *Comoviridae*, especially CPMV, have become a major focus of research in biotechnology. CPMV infects legumes and *Nicotiana benthamiana*, and grows to extremely high titres (>1 g kg^−1^ of leaf tissue). Like many plant viruses, it is also exceptionally stable in the environment. CPMV capsids can now be both genetically and chemically manipulated, and insertion of foreign sequences into surface loops is routine^13^,^14^,^15^. CPMV is therefore being developed for many biotechnology applications, including biosensors, targeted nano-containers for drug delivery, nanomaterials, imaging agents and as a platform for novel vaccine development^16^,^17^,^18^,^19^,^20^,^21^,^22^,^23^,^24^,^25^,^26^,^27^. However, wild-type CPMV has evolved to package its genome, reducing the potential for loading with heterologous cargoes. To overcome this problem, an empty virus-like particle (eVLP) technology has been developed. Expression of the precursor of L and S subunits (VP60) in plants using an Agrobacterium-based, pEAQ vector system^28^ (Fig. 1a) produces large quantities of essentially insoluble protein^12^. Particle formation only occurs on co-expression with the viral 24K proteinase, allowing normal proteolytic processing of VP60 (ref. 12). The result is efficient release of mature coat protein subunits and large quantities of eVLPs, which can be loaded with metal and metal oxide provided the C-terminal residues of the S subunit are first proteolytically removed^13^. Recently, modification of the eVLP surface and particle loading with metal ions (Co^2+^, Fe^2+^ and Fe^3+^) have been achieved simultaneously for the first time^29^, and this may herald attempts to develop targeted therapeutics. Furthermore insertion of integrin-binding sequences into the surface loops of an eVLP has allowed targeting of eVLPs to human endothelial cells (Meshcheriakova *et al*., unpublished).

However, despite much research on CPMV, its true potential in biotechnology may not be realized until we achieve a full understanding of the mechanisms that underlie capsid assembly and encapsidation of genomic RNA (or other cargo). It is not yet, for example, possible to package heterologous RNA or DNA. To gain insights into CPMV biogenesis, we have determined high-resolution cryo-electron microscopy (cryo-EM) structures for the CPMV eVLP, in which the C-terminal extension to the S subunit is visible, to 3.0-Å resolution. We have also determined the structure for wild-type CPMV containing its larger genomic RNA at 3.4 Å resolution, where density for genomic RNA is resolved. The resolution of both maps is sufficient to allow *de novo* atomic models to be built. Furthermore, the availability of a system for producing capsids in the absence of infection has enabled us to use mutagenesis to explore the role of those amino acids predicted by the models to be involved in particle formation, and to discriminate between these and residues involved in RNA packaging. This combined analysis has enabled us to develop a new model for RNA recognition and capsid assembly.

## Results

### Cryo-EM structure determination

**Figure 2:**
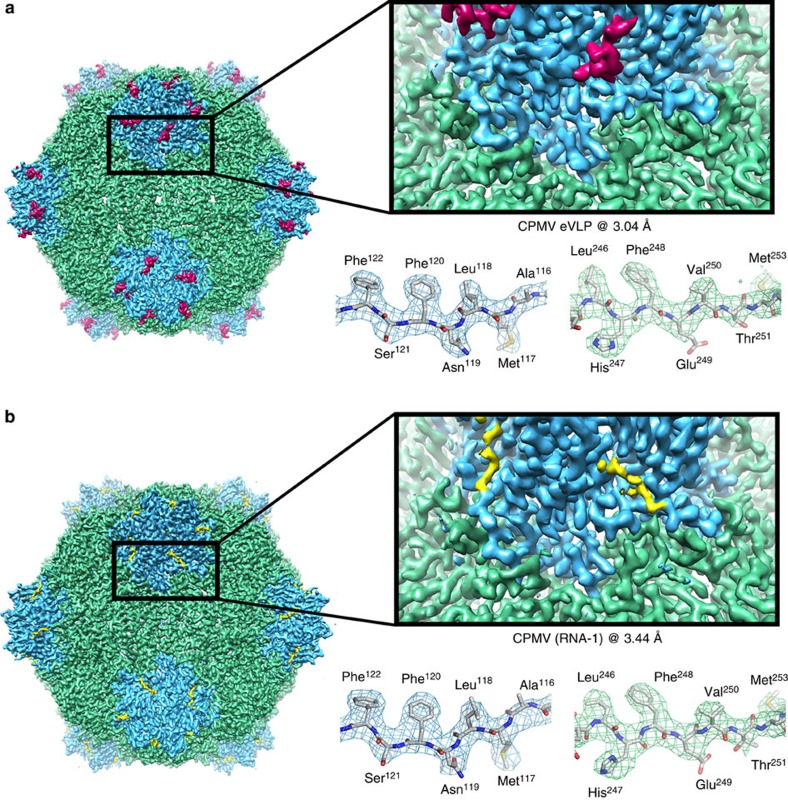
Cryo-EM structures of eVLP and CPMV-B. (**a**) EM density map of CPMV empty virus-like particle (eVLP) determined by cryo-EM to 3.04 Å resolution (EMDB-3014). The L subunit is shown in green, the S subunit in blue and the additionally visualized 13 amino acids in the C-terminal region of the S subunit in magenta. On the right hand side, a zoomed-in view of the boundary between L and S subunits is shown. The density for an individual β strand is shown in a mesh representation with the EM-derived atomic model within, showing clear resolution of large and small side chains. (**b**) Identical views as in **a**, but showing the EM map of CPMV containing RNA-1 (CPMV-B) to 3.44 Å resolution (EMDB-3013).

Although the C-terminal extension to the S subunit is implicated in capsid assembly and RNA packaging, understanding these roles has been difficult because the normal maturation of the RNA-filled capsid involves its cleavage and dissociation. As a result no structural information for these residues is currently available. To address this deficiency, we decided to examine the structure of the CPMV eVLP particle. Crucially, not only are such eVLPs completely lacking in any encapsidated RNA, they also undergo C-terminal cleavage more slowly than wild-type virions, raising the possibility that we could determine the structure of an eVLP that retains the C-terminal segment using cryo-EM and single particle image processing. We therefore collected a cryo-EM data set for CPMV eVLP comprising ∼1,150 micrographs collected on an FEI Titan Krios microscope, using a direct electron detector (for details of data collection and image processing see Methods). Each micrograph was recorded as an exposure movie consisting of 35 frames, which were computationally corrected for microscope drift and beam-induced movement^30^. Particles were selected semi-automatically^31^, and a data set of 62.5k particles was assembled. Iterative rounds of two-dimensional (2D) and 3D classification were then used to select a homogeneous subset of 4,998 particles for 3D structure refinement (see Methods). The resulting final density map was sharpened using an empirically derived *B*-factor of −74.6 Å^2^ to 3.04 Å resolution (Fig. 2a; EMD-3014).

Wild-type, infectious CPMV particles containing RNA-1 (bottom fraction; CPMV-B) were collected from the bottom of a Nycodenz gradient, dialysed to remove the Nycodenz, and used for cryo-EM data collection. A data set of ∼1,750 electron micrographs was collected on the same microscope and detector as described above. Particles were selected automatically, generating a total data set of ∼72k particles. A homogeneous subset (4,331) of these particles was selected and used to determine a 3D reconstruction. The final structure for CPMV-B was sharpened using an empirically derived *B*-factor of −107.6 Å^2^ to a final resolution at 3.44 Å (Fig. 2b; EMD-3013). It should be noted that the initial starting model for the eVLP structure was a sphere with a radius of ∼155 Å. The CPMV-B structure used the eVLP model filtered to 60 Å resolution. No information from the existing X-ray structure whatsoever was therefore used to generate either of the structures presented here.

### Atomic model building

As shown in Fig. 2, the resolution of both eVLP and CPMV-B maps is high enough to clearly resolve amino acid side chains in the density. We therefore decided to build *de novo* atomic models into the EM density rather than rely on existing atomic models for the CPMV capsid proteins (PDB 1NY7^4^). We started with the higher resolution eVLP map, and built the polypeptide chain of a single copy of both the L and S subunit using Coot^32^. This preliminary model was then iteratively refined and rebuilt using REFMAC5^33^ and Coot^32^ to progressively improve model quality. The resulting model contained information for the majority of the polypeptide sequence, critically including a 13-residue segment in the C-terminal region of S subunit that had never been previously visualized. The refined eVLP atomic model was then docked into the 3.4-Å CPMV-B map. Residues in the eVLP atomic model for which no density was observed for CPMV-B were deleted (residues 190–202 in S subunit) and amino acids resolved in CPMV-B but not eVLP were added and modelled (residues 184–189 in the S subunit). This preliminary (for the CPMV-B structure) model was then again iteratively refined in REFMAC5 to give the final model presented in Fig. 2b.

### The structure of the C-terminal extension to the S subunit

**Figure 3:**
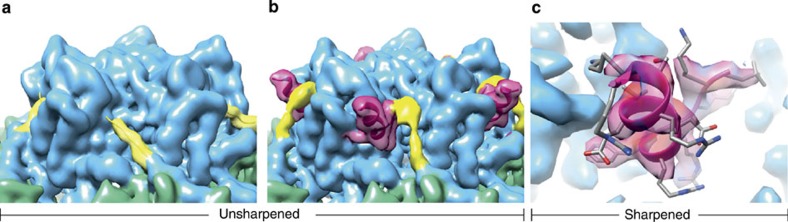
The structure of the C-terminal extension of the S subunit. (**a**) EM density map of the unsharpened CPMV-B map with colours as described previously. In yellow is the C terminus of CPMV-B (amino acids 184–189), which follows the same path as the current atomic model (PDB 1NY7^4^). The final C-terminal amino acids (190–213) are missing from both the crystal structure and the CPMV-B EM density map. (**b**) EM density map of the unsharpened eVLP map with colours as described previously. The density corresponding to amino acid 184–189 is coloured yellow. Although this section of the EM density is too weak to allow a polypeptide backbone to be built, we can clearly see this portion of the C-terminal moves in the eVLP map compared with the CPMV-B map (see yellow segment in **a**). Coloured magenta is the newly resolved 13-amino acid residue (190–202 in the S subunit). (**c**) Zoomed-in version of the C-terminal extension from the sharpened EM density. The new atomic model is shown inside.

The existing structural information for the CPMV capsid^3^,^4^ show the C terminus of S subunit after cleavage (ending at residue 189) in an extended conformation running across the exterior surface of the capsid towards a cleft between the S subunits that form the turret at an icosahedral fivefold vertex. This is precisely the conformation we see in our CPMV-B structure (see the yellow segment in Fig. 3a), but in the eVLP map we see additional density in this cleft that does not match the previously deposited structure. The density that would correspond to residues 184–189 in the C terminus is very weak suggesting this segment is poorly ordered in the particle in solution (see the yellow segment in Fig. 3b), and we have not been able to build a convincing model into this region of the map. However, it is clear that the polypeptide chain takes a steeper path along the edge of the cleft than it does once C-terminal cleavage (between residues 189 and 190) has occurred (comparison of yellow segments in Fig. 3a,b). The C-terminal segment then becomes ordered once more, and we see density corresponding to Leu190 to Arg202, residues absent from previous structures. A loop runs from the top of the S subunit back into the cleft between subunits, before forming two turns of α-helix running out of the cleft towards the bulk solvent (see magenta segment Fig. 3b). The bottom of this segment appears to be very well-ordered, with clear density for side chains that make intimate contacts to the neighbouring S subunit around the penton (Fig. 3c). The density then becomes disordered once more, with Arg202 as the last ordered residue, suggesting that the 11 C-terminal residues are disordered in solution. Intriguingly, this tallies with functional observations that while truncation of the C-terminal segment by up to 11 residues are tolerated, larger truncations (12 residues or more) dramatically reduce the yield of intact eVLPs (see Supplementary Fig. 1 and Supplementary Table 1).

### The role of the C-terminal extension

**Figure 4:**
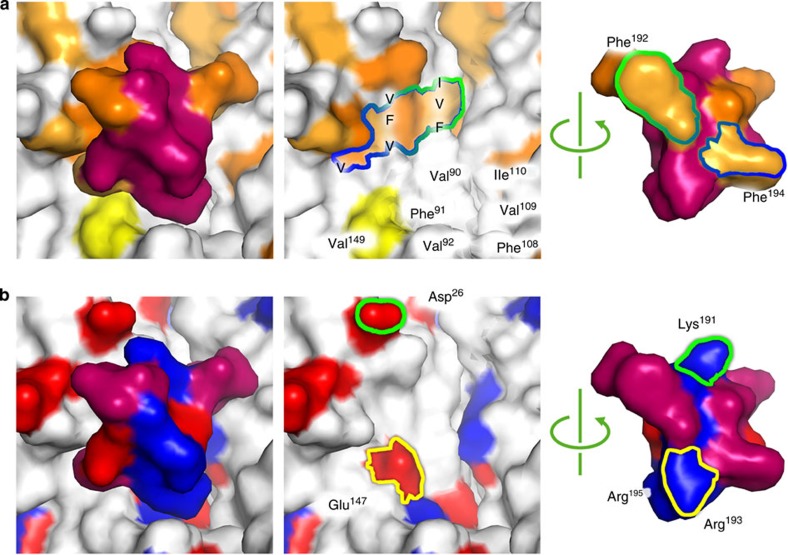
Interactions between the C-terminal extension and the neighbouring S subunit. (**a**) EM-derived atomic model for the eVLP S subunit and C-terminal extension represented as a surface model and coloured according to hydrophobicity (I, L and V: orange; G, A and F: pale orange; C and M: yellow). The middle panel shows the surface of the S subunit with the C-terminal extension removed, and the right panel shows the surface of the C-terminal extension that interacts with the S subunit. Hydrophobic residues are labelled. (**b**) As in **a** coloured according to charge (red is negative, blue is positive). The charged residues from S subunit are labelled. All residues in Fig. 4 are from the S subunit.

**Figure 5:**
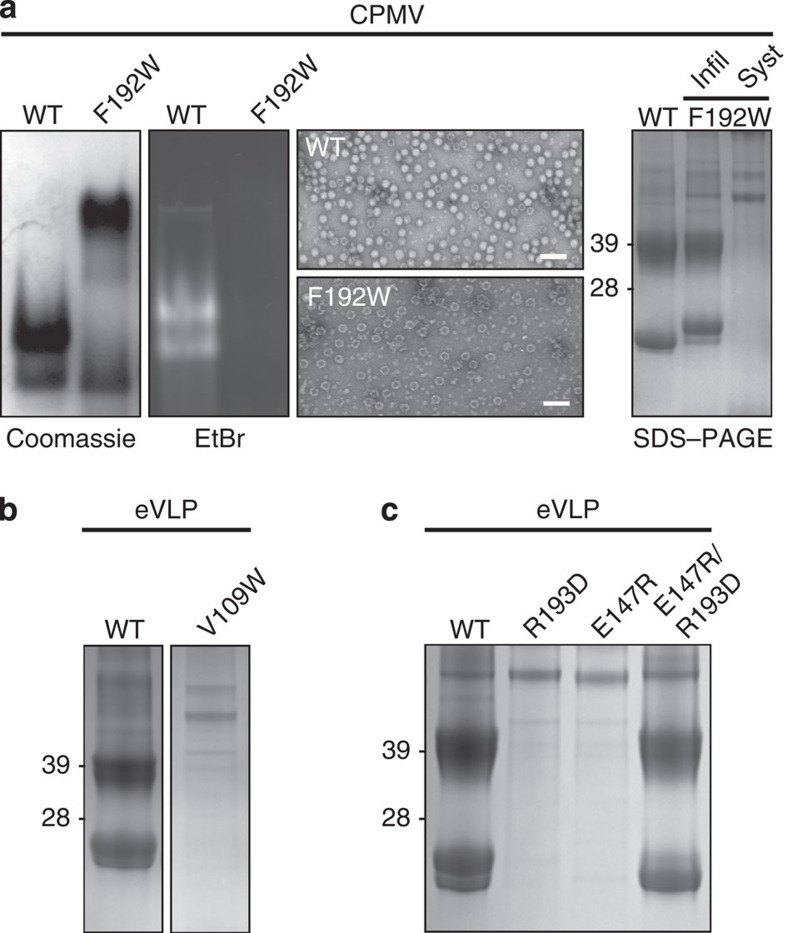
Residues in the S subunit that are important for particle assembly and genome encapsidation. (**a**) Agarose gels stained with either Coomassie blue or ethidium bromide (EtBr) show that the F192W mutant packages no RNA. Negative stain EM illustrates ‘empty' particles in F192W mutant compared with WT. Scale bars, 100 nm. SDS–PAGE shows that the level of protein expression is comparable in F192W compared with WT in infiltrated leaves; however, F192W is unable to cause a systemic infection. (**b**) SDS–PAGE showing that the V109W mutation abolishes the assembly of eVLP. (**c**) SDS–PAGE showing R193D and E147R mutations also prevent eVLP assembly; however, the double mutation of E147R/R193D, which preserves the salt-bridge, permits very similar levels of capsid assembly compared with WT.

The ordered C-terminal segment described for the first time here forms an intimate network of interactions with the neighbouring S subunit around the pentameric ring that forms the fivefold vertex of the particle. It is clear from the structure that hydrophobicity plays a central role in this network. Shown in Fig. 4a is the EM-derived atomic model for the eVLP represented as a surface, and coloured according to the hydrophobicity of the corresponding amino acid residues involved (see legend of Fig. 4 for details). Two phenylalanine residues in the C-terminal segment (F192 and F194) are well-resolved and appear to bind to a large hydrophobic patch on the body of the neighbouring S subunit. To test the importance of these interactions, we made mutations in the S subunit sequence and analysed their effects on both eVLP assembly and RNA packaging by the virus. While F192W has little discernable effect on eVLP assembly, it dramatically reduces the efficiency of RNA packaging, resulting in large numbers of empty capsids and systemic movement of the virus in the plant does not occur (Supplementary Table 2 and 3; Fig. 5a). Mutation of the matching hydrophobic surface on the S subunit itself (for example, V109W) has even more profound effects, preventing assembly of particles (Fig. 5b). However, the network of interactions is complex, as mutation of the other phenylalanine residue (for example, F194W) has little discernable effect other than a slightly reduced particle yield.

Despite the extensive nature of the hydrophobic surface on both C-terminal extension and the S subunit surface to which it binds, a number of charged residues also appear to play a key role. The C-terminal segment is highly basic, and side-chain density for two arginine residues is visible at the bottom of the cleft (R193 and R195; see Fig. 4b). R193 is particularly well-ordered and forms a salt-bridge to E147, again in the neighbouring S subunit. To test the importance of this interaction, we mutated these residues and assayed for eVLP assembly and viral encapsidation of RNA *in vivo*. Both R193D and E147R are completely unable to assemble, while the double mutant R193D/E147R, which preserves the salt-bridge but swaps its directionality, is almost indistinguishable from the wild type (Fig. 5c). R195G is similar to wild-type in terms of assembly, suggesting that it is the R193-E147 salt-bridge that is crucial for assembly (Supplementary Table 2).

### Interactions between the protein capsid and genomic RNA

The way in which eVLPs are expressed means that no genomic RNA is present in the cell, so none can possibly be packaged. However, the eVLP has previously been shown to be devoid of plant cell mRNA^13^, including the recombinant message for either the viral coat proteins or proteinase, which are the two mRNAs that should have the highest sequence similarity to the genome^12^. Indeed, in the 3.0 Å structure of the eVLP there is no EM density that can be attributed to anything other than capsid proteins, which together with their very low A260/280 ratio and lack of ethidium bromide staining in agarose gels^13^ strongly suggest that the eVLP particles are devoid of RNA.

**Figure 6:**
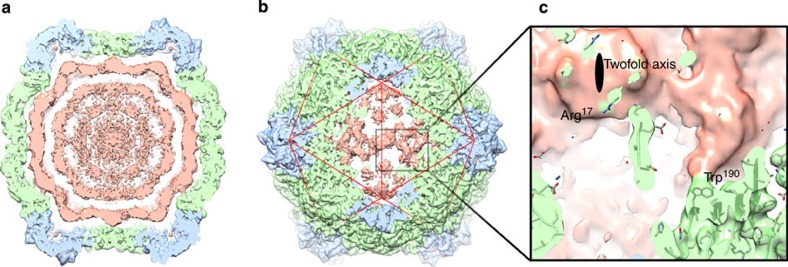
Density for RNA-1 in the CPMV-B structure. (**a**) A 40-Å thick central slab through the unsharpened CPMV-B map (at 3.63 Å resolution; unsharpened map is also included in the deposition for EMDB-3013, suggested contour level 0.015). The extra density ascribed to RNA is pink. Viral coat proteins coloured as described previously. (**b**) The strongest density for RNA is found beneath the capsid twofold axis, a binding site formed at the interface between two adjacent pentons. (**c**) Close up of viral RNA–protein interactions, demonstrating two major bridges of density between the viral RNA and the protein capsid. The density bridges are consistent with the involvement of W190 and R17 (both from the L subunit) in RNA binding.

By contrast, the wild-type CPMV-B particle has packaged the full-length, 6-kb single-stranded RNA-1, and as expected we see significant extra density inside the capsid that we ascribe to this packaged RNA genome. However, the *B*-factor correction used to sharpen the map and reveal high-resolution features such as amino acid side chains acts as a strong high-pass Fourier filter, removing low-resolution features in the map such as poorly ordered molecular components like the genomic RNA. Shown in Fig. 6a is a 40-Å-thick central slab through the unsharpened CPMV-B map (at 3.63 Å resolution; the unsharpened map is also included in the deposition for EMDB-3013). As seen in the cryo-EM structures of several single-stranded RNA viruses^34^,^35^, the RNA appears as concentric shells of density. It must be noted that this density is an icosahedrally averaged picture of an asymmetric RNA molecule, so precise structural interpretation is impossible. However, several observations can be made. First, the shells of density have a thickness of ∼20 Å, and are ∼20–25 Å apart, consistent with the packing of duplex nucleic acids observed in other virus structures (for example, refs 34, 36), suggesting that extensive base pairing occurs during encapsidation. The general form of the packaged RNA is dodecahedral rather than icosahedral (each is a different realization of 532 symmetry), with the strongest RNA feature in the map forming a truncated dodecahedral cage beneath the capsid shell. This is strongly reminiscent of RNA packaging in insect viruses of the *Nodaviridae*, where ordered genomic RNA is packaged as a dodecahedral cage in both X-ray and EM structures^37^,^38^. The strongest CPMV density is directly beneath the twofold symmetry axes of the capsid, which are formed by the interface between two adjacent pentons, implying that this is the site where RNA binding is strongest. The density fades out as the RNA extends away from the twofold axis towards the threefold junctions that form the vertices of the truncated dodecahedron. We see two major bridges of density between the capsid shell and the RNA density that are candidates for amino acid side chains, both from the L subunit, that directly interact with RNA (Fig. 6c). These are Arg17 and Trp190, with Trp190 being by far the strongest density feature connecting the capsid shell to the RNA.

**Figure 7:**
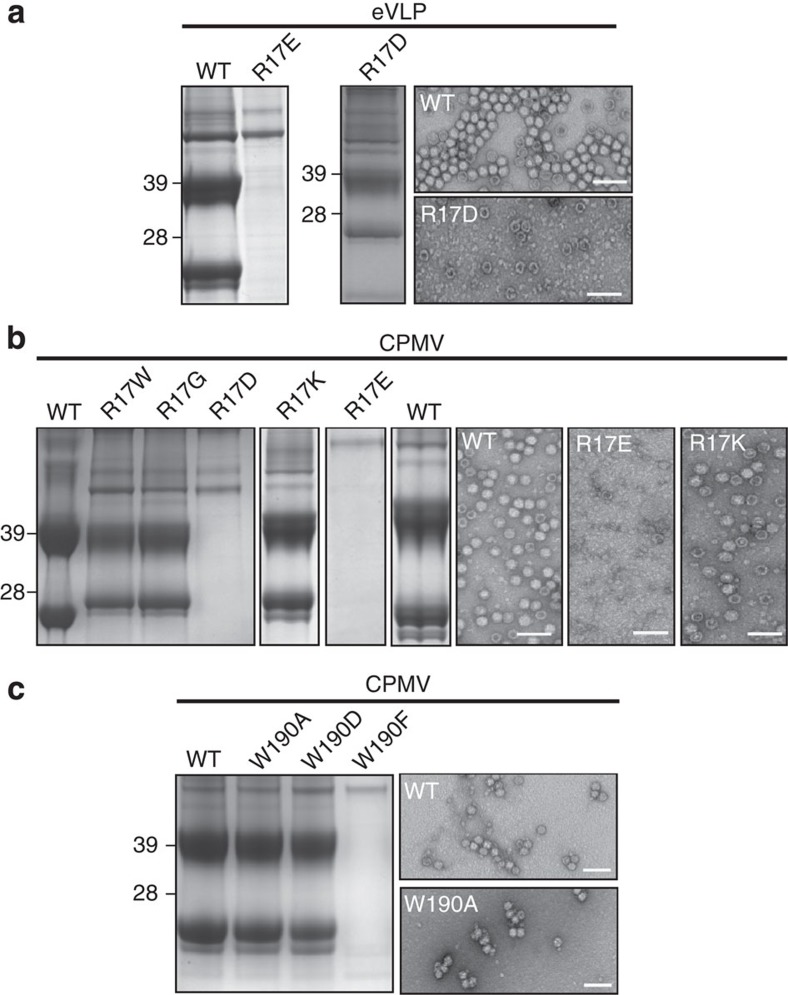
Residues involved in encapsidation of genomic RNA. (**a**) SDS–PAGE and negative stain EM showing the R17D mutation in eVLP has little effect on capsid assembly, however, R17E completely abolishes it. EM images of the WT eVLP show different stain penetration depending on the presence or absence of the C terminus, with stain failing to penetrate complexes with an intact C terminus. The preparation in the EM is completely RNA free. (**b**) SDS–PAGE and negative stain EM showing R17 mutants in the L subunit. R17D and R17E abolish capsid assembly. Some flexibility is, however, tolerated as R17W, R17G and R17K all behave similarly to WT virus. (**c**) SDS–PAGE and negative stain EM showing W190 mutants in the L subunit. W190F abolishes RNA binding and particle assembly. However, W190A and W190D are indistinguishable from WT. All scale bars, 100 nm.

To test the importance of these residues, we mutated each and examined the effect on eVLP assembly and RNA encapsidation *in vivo* (Fig. 7). Arg17 does indeed appear to be important for RNA packaging. Although R17D can be introduced into eVLPs with little effect, R17E substantially reduces eVLP capsid assembly (Supplementary Table 2; Fig. 7a). In wild-type virus, R17E and R17D abolish RNA packaging and substantially reduce capsid assembly (Supplementary Table 3; Fig. 7b). R17W, R17G and R17K are all indistinguishable from wild-type virus (Supplementary Table 3; Fig. 7b), with identical yield and systemic transport in plants, suggesting that the some degree of flexibility in the nature of the residue is tolerated. Similarly, mutations of W190A or W190D are both indistinguishable from wild-type virus, while W190F abolishes both RNA binding and capsid assembly (Supplementary Table 3; Fig. 7c).

## Discussion

The cryo-EM structures presented here provide new insights into the structure of viruses in the *Comoviridae*. They demonstrate the ability of cryo-EM structures to guide site-directed mutagenesis that can begin to pick apart the molecular mechanisms that govern capsid assembly and genome packaging, providing the basis for a new model of CPMV particle assembly.

**Figure 8:**
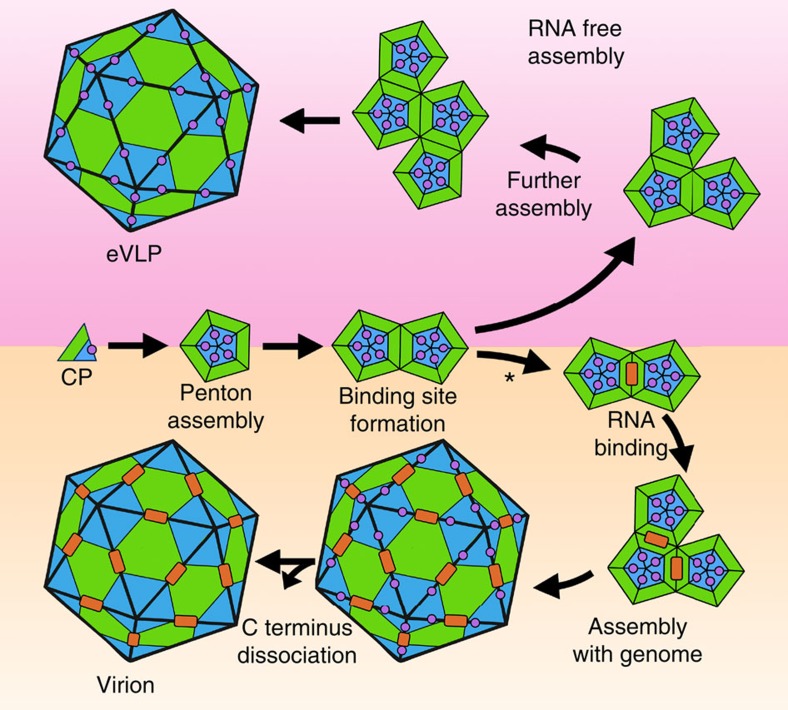
A model for CPMV Assembly. The L (green) and S (blue) subunits associate to form a coat protein penton. The C-terminal extension (magenta circle) appears to act as a dab of ‘molecular glue', stabilizing the formation of the penton due to extensive interactions with the neighbouring S subunit. In the absence of RNA (RNA free assembly shown in the top half of the diagram), C-terminal cleavage is slow, and eVLP particles are produced. After time, the C-terminal extension is lost from the eVLP, suggesting that once an extensive protein:protein interaction network is formed in the capsid the eVLP structure is stable. In the presence of genomic RNA (assembly with genome shown in the bottom half of the diagram), assembly follows a different path. Initially, pentons are formed in the same way as in the eVLP, using the C-terminal extension for stability. Two pentons interact to form a twofold axis, and thus the RNA-binding site appears to exist at that position. Our model shows genomic RNA (represented as an orange rectangle) initially binds to the two pentons at this twofold axis, the first step in genome encapsidation. Following stepwise addition of pentons, the C terminus is rapidly cleaved implying that RNA occupancy is allosterically communicated through the structure to the outside of the capsid.

The C-terminal region of the S subunit, despite its importance for particle formation, is normally lost during virus maturation. The novel structure of this region presented here therefore reveals for the first time an extensive protein:protein interface that combines both hydrophobicity and electrostatic interactions that are important for capsid assembly and RNA encapsidation. Many questions remain, but the C-terminal extension occupies the cleft between the small coat protein subunits that form the pentameric capsomere from which the virus assembles (Fig. 8). While we do not resolve the residues that connect the C-terminal structure to the body of the S subunit (residues 184–189), we do see extensive interactions to the neighbouring subunit around the pentameric ring. The C-terminal extension may act therefore as a dab of ‘molecular glue' stabilizing the structure of the penton of L and S subunits. In the absence of genomic RNA, this stabilization appears to be sufficient to allow formation of the intact eVLP. The eVLP does lose its C-terminal extension over time, implying that the stability gained from making the many protein:protein interactions formed during capsid assembly is sufficient to make the eVLP structure stable. In this context, the role of the C-terminal extension would in fact precisely fit the definition of a molecular chaperone or a scaffold protein, enhancing the efficiency of folding/assembly reactions without becoming a part of the final structure.

In the presence of genomic RNA, however, assembly takes a very different path. If the C terminus stabilizes the CPMV penton, then the next step in building a capsid is the interaction between two pentons, which forms the first twofold axis of the growing particle. This is precisely the position at which our EM density suggests there is the strongest interaction with the genomic RNA. We suggest therefore that the C-terminal extension promotes the formation of the RNA-binding site itself, the first step in encapsidating the genomic RNA (Fig. 8; see asterisk). This is in contrast to previous models of BPMV assembly where RNA binding is proposed to occur at particle threefold axes^39^. However, the rapid cleavage of the C terminus in such conditions suggests that RNA binding must be allosterically communicated through the structure to the binding site of the C terminus on the outside of the capsid. Furthermore, the lack of obvious mixed electrophoretic forms that would reveal a mixture of cleaved/uncleaved subunits suggests that cleavage is strongly cooperative within a particle. The precise details of this communication remain unresolved, but the network of interactions is clearly complex. Mutation of residues on the inside of the capsid that directly interact with RNA prevent capsid assembly (for example, W190F in the large subunit). Conversely, residues on the outside of the capsid that were predicted to disturb protein:protein interactions allow capsid assembly but prevent RNA encapsidation (for example, F192W). Further structural studies on these mutants will likely help to elucidate the fine detail of these interactions.

All members of the *Comoviridae* have a similar, cleavable C-terminal extension to their small coat protein subunit, although its length and sequence are highly variable. For example, in BPMV the sequence is strongly negatively charged in contrast to the positive charge found in CPMV. The structures presented here help to explain this diversity, because sequence changes in the C-terminal extension could easily be compensated for by surface mutations on the small subunit itself, as we show in the viability of the R193D/E147R mutant, which makes CPMV more BPMV-like in this regard. This idea is also consistent with observations that replacing the CPMV extension with the equivalent region from BPMV results in a virus with similar properties to one from which the C-terminal residues have been deleted^11^.

Although our studies provide an excellent view of capsid–RNA interactions in assembled particles, they do not, as yet, provide insights into how the specificity of RNA packing is achieved; this remains a goal of future structural studies in this system. The prevailing view in *Picornavirales* research is that RNA encapsidation is tightly linked to RNA replication. The paradigm is thus that specificity is a function of protein:protein rather than protein–RNA interactions, and that nucleotide sequence specificity is apparently unimportant. Our finding that the C-terminal 24 amino acids of the S subunit are involved in stabilizing the coat protein penton fits into this paradigm. The stabilization of the penton and the resulting promotion of interactions between pentons explains how this region can promote RNA encapsidation despite this sequence lying on the external surface of the assembled particle. Similar sequences are not found in other plant RNA viruses, perhaps because they commonly interact with their genomes via charged N-terminal arms that are missing in the comoviruses. However, conceptually the role of the C-terminal sequence may be similar to the effect seen in Hepatitis A, an animal picornavirus, where the uncleaved 2A peptide at the C terminus of VP1 strongly promotes capsid assembly^29^. It also offers an explanation as to why this sequence can act to suppress RNA silencing when part of the S subunit^11^ but not when it is fused to other proteins such as green fluorescent protein (Cañizares and Lomonossoff, unpublished), as the C-terminal extension's role is in chaperoning the formation of the silencing-active surface rather than silencing itself. However, given that the presence of the RNA accelerates the rate of C-terminal cleavage, it is plausible that both the C terminus and the genomic RNA may co-chaperone the assembly of the nascent virus particle.

## Methods

### CPMV-B and CPMV eVLP purification

Infection of *N. benthamiana* with CPMV was initiated by agroinfiltration of 3-week-old plants with pBinP-S1NT and pBinP-S2NT^40^. Wild-type eVLPs were produced either by co-infiltration of 3-week-old *N. benthamiana* with pEAQ-*HT*-VP60 and pEAQ-*HT*-24K^12^ or by infiltration with the single plasmid, pEAQexpress-VP60-24K^15^. Leaves were harvested 5–7 days post infection. Wild type CPMV^41^ and eVLPs^29^ were extracted and purified (described below).

CPMV and eVLP particles were purified by the following method; leaf tissue was homogenized in a blender in 3 volumes of 0.1 M sodium phosphate buffer, pH 7.0. The homogenate was filtered through two layers of muslin and clarified by centrifugation at 13,000*g*, for 20 min at 4 °C. Virus particles were precipitated using 0.25 volumes of 20% (w/v) PEG6000 and 1 M NaCl and pelleted by centrifugation at 13,000*g* for 20 min at 4 °C. The resulting pellet was resuspended thoroughly in 10 mM sodium phosphate, pH 7.0. eVLPs were purified further by centrifugation at 27,000*g* for 20 min at 4 °C followed by a centrifugation of the clarified supernatant at 118,700*g* for 2.5 h at 4 °C. The pellet was resuspended in small volume of 10 mM sodium phosphate buffer overnight at 4 °C. The eVLP suspension was further purified by centrifugation at 16,000*g* for 15 min at 4 °C. For wild-type CPMV and mutated CPMV, the individual components were separated by centrifugation on 30–60% (w/v) Nycodenz gradients^42^.

### Mutagenesis

Point mutations were introduced into the coat protein-coding region of either pEAQ-*HT*-VP60 or pEAQ-RNA-2, a plasmid containing a full-length copy of CPMV RNA-2 in pEAQ-*HT* using the GENEART Site-Directed Mutagenesis System (Invitrogen) according to manufacturer's protocol. Primers for site-directed mutagenesis were designed using QuikChange Primer Design Program (Supplementary Table 4). To produce eVLPs containing the required mutation(s), the mutant forms of pEAQ-*HT*-VP60 were infiltrated into *N. benthamiana* in the presence of pEAQ-*HT*-24K^12^ and mutant eVLPs, if present, were extracted. To examine the effect of the mutations on virus infectivity and RNA encapsidation, the mutant forms of pEAQ-RNA-2 were co-infiltrated into *N. benthamiana* with pBinP-S2NT and any particles produced were purified as described for wild-type CPMV (above).

Deletions in the C-terminal region of the S subunit of eVLPs were made by PCR amplification using pEAQ-*HT*-VP60 as a template^12^. The primers used for PCR also contained appropriate restriction sites (BspEI and StuI) to replace the full-length VP60 with deleted variants of S subunit in pEAQ-*HT*-VP60 (Supplementary Table 5). The resulting deletion mutants were verified by sequencing before agroinfiltration of *N. benthamiana* plants in the presence of pEAQ-*HT*-24K. The formation of assembled capsids was assessed as described above. Raw images of uncropped gels are shown in Supplementary Figs 2 and 3).

### Grid preparation and imaging

Cryo-EM grids were prepared by placing 3 μl of ∼4.2 mg ml^−1^ (eVLP) or ∼5.8 mg ml^−1^ (CPMV-B) onto 200 mesh grids with 2-μm holes (Quantifoil R2/2, Quantifoil Micro Tools, GmbH, Germany). Grids were glow discharged for ∼20 s prior plunge freezing in liquid ethane cooled by liquid nitrogen, using a FEI Vitrobot IV at 100% relative humidity. Data was collected on an FEI Titan Krios (MRC-LMB, Cambridge, UK.) transmission electron microscope at 300 kV, using an electron dose of ∼45e^-^ per Å^2^ and a magnification of × 134,615. The final object sampling was therefore of 1.04 Å per pixel. A total of 1,135 (eVLP) and 1,754 (CPMV-B) exposures were recorded using the EPU automated acquisition software on a 17 Hz FEI Falcon II direct electron detector. Each exposure movie had a total exposure of 2 s and contained 35 images.

### Image processing

Drift-corrected averages of each movie were created using MOTIONCORR^30^ and the contrast transfer function of each determined using CTFFIND3 (ref. 43); any images showing signs of astigmatism were discarded. All subsequent image processing steps were performed using RELION (v1.3)^44^ unless otherwise stated. Approximately 1,000 particles were manually picked and classified using reference-free 2D classification. The resulting 2D class average views were used as templates for automated particle picking^31^ (see Table 1 for particle numbers at each processing step). To produce a structurally homogeneous subset of CPMV particles, this initial stack was reduced using a statistical particle-sorting algorithm^31^, which excludes the particles that are least similar to the initial search references. The remaining particles were classified using reference-free 2D classification to yield a data set for 3D structure refinement that only includes isolated molecular views of CPMV (see Table 1). These data sets, which comprise ∼80% of all initially selected particles, were used to calculate initial 3D reconstructions at ∼3.5 Å (eVLP) and 4.1 Å (CPMV-B). We then searched within each data set for a subset of particle images with greater homogeneity and/or higher resolution, using sequential 3D classification steps with icosahedral symmetry imposed. Each of these steps split the data into two, and the class with the sharpest features and highest resolution was taken forward. The initial starting model for the eVLP structure was a sphere with a radius of ∼155 Å. For the CPMV-B structure, the eVLP structure filtered to ∼60 Å resolution was used. Following 7 rounds of 3D classification for eVLP and 13 rounds for CPMV-B, the final data sets (consisting of ∼8–10% of the original data) were considered to be homogeneous and used to determine final 3D reconstructions^45^. To correct for mechanical drift, beam-induced movement and radiation damage, statistical movie processing and particle polishing procedures were implemented^45^. As CPMV particles are readily visible even in individual movie frames, a running average of three frames was used in the calculations. Post-processing was employed to appropriately mask the model, estimate and correct for the *B*-factor of the maps^46^. The final resolution was determined using the ‘gold standard' Fourier shell correlation (FSC=0.143) criterion^44^ as 3.04 Å for eVLP and 3.44 Å for CPMV-B (FSC curves are shown in Supplementary Fig. 4). Local resolution was estimated using the ResMap wrapper in RELION^47^.

### Model building and refinement

The backbone of each polypeptide chain in a single asymmetric unit of the 3 Å eVLP cryo-EM map was built *de novo* using the ‘baton building' tool in Coot^32^. The sequence was manually entered and the model refined using the ‘real space refinement tool' in Coot^32^. Prior to refinement, secondary structure restraints were applied using ProSMART^48^. Structure factors obtained from the Fourier transform of the experimental density were used to restrain the coordinates in REFMAC5 (ref. 33). After each refinement round non-ideal rotamers, bond angles and Ramachandran outliers were improved. The final model was assessed for quality using MolProbity^49^. The refined eVLP atomic model was docked into the 3.4 Å CPMV-B map. Residues in the eVLP atomic model for which no density was observed in the CPMV-B map were deleted (residues 190–202 in S subunit) and amino acids resolved in CPMV-B but not eVLP were added and modelled (residues 184–189 in S subunit). External restraints (produced from the eVLP model) and secondary structure restraints were applied using ProSMART, the structure was refined in REFMAC5 and assessed for quality using MolProbity (Table 2). Figures were generated using Chimera^50^ and PyMOL^51^.

## Additional information

**Accession codes:** Coordinates are deposited in the Protein Data Bank under accession code 5A33 for CPMV eVLP and 5A32 for CPMV-B. Cryo-EM reconstructions are deposited in the EM Data Bank under accession codes EMD-3014 CPMV eVLP and EMD-3013 for CPMV-B.

**How to cite this article:** Hesketh, E. L. *et al*. Mechanisms of assembly and genome packaging in an RNA virus revealed by high-resolution cryo-EM. *Nat. Commun*. 6:10113 doi: 10.1038/ncomms10113 (2015).

## Supplementary Material

Supplementary Supplementary Figures 1-4 and Supplementary Tables 1-5

## Figures and Tables

**Figure 1:**
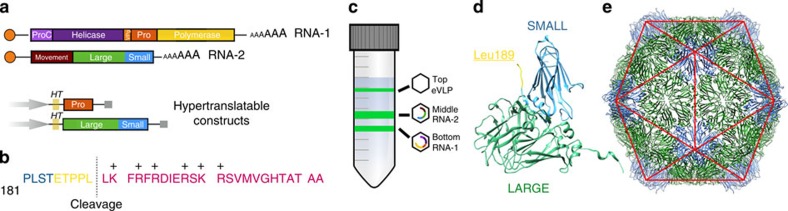
An introduction to CPMV. (**a**) Schematic of the Cowpea mosaic virus (CPMV) bipartite, positive-sense single-stranded RNA genome. RNA-1 is 6 kb in length and contains the non-structural genes, while RNA-2 is 3.5 kb in length and contains the sequence encoding the large coat protein (L subunit) and small coat protein (S subunit). Hypertranslatable constructs (*HT*) separately expressing the viral proteinase and the precursor of the L and S subunits are used for production of eVLPs using the pEAQ vector system. (**b**) The sequence of S subunit amino acids 180–213. The C-terminal 24 amino acid segment of S subunit is cleaved following assembly, and is coloured magenta. The cleavage site between Leu189 and Leu190 is shown. This region of the polypeptide is highly positively charged and the positive amino acids are indicated. (**c**) Schematic of CPMV density purification. RNA-1 containing CPMV sediments at the bottom of a Nycodenz gradient (CPMV-B), RNA-2-containing CPMV (CPMV-M) sediment in the middle and empty CPMV particles sediment at the top of the gradient (CPMV-T). CPMV-T particles are the natural equivalent to empty virus-like particles (eVLPs). (**d**) X-ray crystal structure of the asymmetric unit of CPMV (PDB 1NY7^4^), coloured as above. The C-terminal amino acid of the S subunit (leucine 189) is indicated. (**e**) Icosahedral organization of CPMV (using PDB 1NY7^4^). Each icosahedral particle is comprised of 60 copies of both the L subunit and the S subunit. A view down the twofold axis is shown.

**Table 1:** Particle numbers at each stage in image processing.

	eVLP	CPMV-B
Autopicking	62,514	72,061
Particle sorting	52,970	65,346
2D classification	39,800	48,189
3D classification	4,998	4,331

eVLP, empty virus-like particle; CPMV-B, cowpea mosaic virus B; 2D, two-dimensional; 3D, three-dimensional.

**Table 2:** Refinement and model statistics for atomic models built *de novo* into eVLP and CPMV-B EM density.

	eVLP	CPMV-B
*Data collection*		
Particles	4,998	4,331
Pixel size (Å per pixel)	1.04	1.04
Defocus range (μm)	0.5–5.0	0.5–8.0
Voltage (kV)	300	300
Dose (e^-^ per Å^2^)	45	45
		
*EM refinement*		
Final resolution (Å)	3.04	3.44
Experimental *B*-factor (Å^2^)	−74.6	−107.6
EMDB accession number	EMD-3014	EMD-3013
		
*r.m.s. deviations*		
Bonds (Å)	0.0077	0.0108
Angles (°)	1.2957	1.5319
		
*Model validation*		
R factor	0.2207	0.2909
Molprobity score	1.61 (100th percentile)	1.76 (100th percentile)
Clashscore (all atoms)	0.81 (100th percentile)	1.52 (100th percentile)
Good rotamers (%)	96.3%	96.5%
PDB ID	5a33	5a32

EM, electron microscopy; eVLP, empty virus-like particle; CPMV-B, cowpea mosaic virus B; r.m.s., root mean squared.
