# Supplementary material for: Mechanisms of assembly and genome packaging in an RNA virus revealed by high-resolution cryo-EM
Source: Nat Commun. 2015 Dec 10;6:10113. doi: 10.1038/ncomms10113 (PMC4682053; doi:10.1038/ncomms10113)
Supplement: Supplementary — Figures 1-4 and Supplementary Tables 1-5 [file ncomms10113-s1.pdf]

SUPPLEMENTARY FIGURES

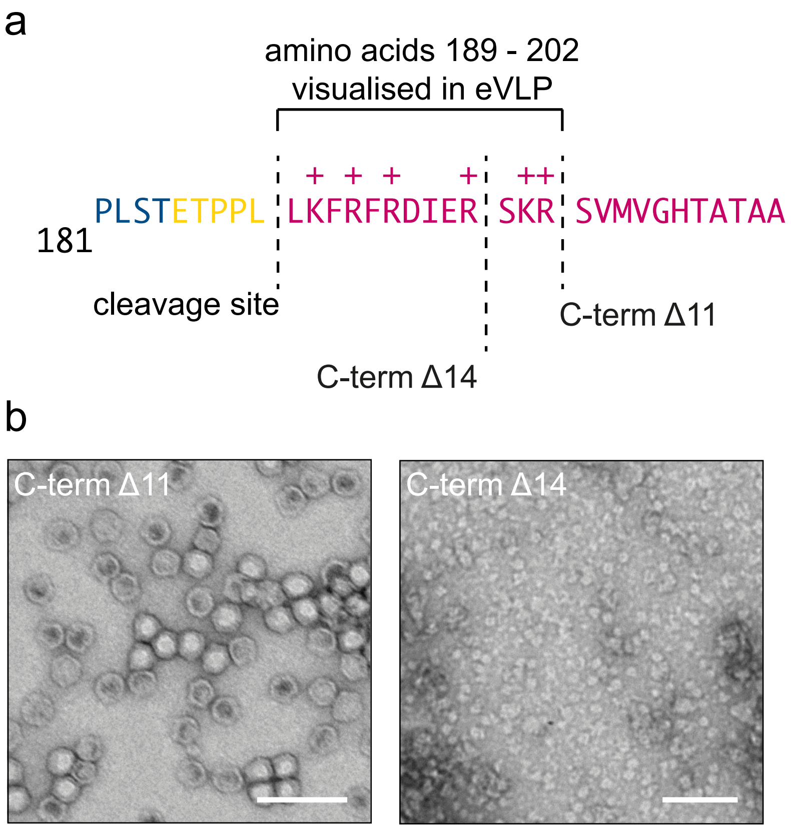

Supplementary Figure 1. CPMV eVLP C-terminal deletion mutants

a. The sequence of S subunit C-terminal amino acids 180 - 213. The C-terminal 24 amino acid segment of S subunit is cleaved following assembly, and is coloured magenta. The natural cleavage site between Leu189 and Leu190 is shown. This region of the polypeptide is highly positively charged and the positive amino acids are indicated. The positions of the C-terminal deletion mutants are also indicated. The C-term Δ11 mutant is missing the C-terminal 11 amino acids and the C-term Δ14 mutant is missing the C-terminal 14 amino acids.

b. Negative stain electron microscopy illustrating the C-term Δ11 mutant can form particles, however the C-term Δ14 mutant does not form particles. Scale bars are 100 nm.

Gels from figure 5a

1 2

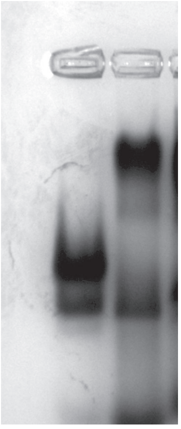

Coomassie

Lane 1 - WT CPMV, Lane 2 - F192W

1 2

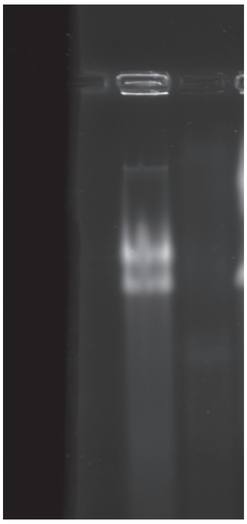

Ethidium Bromide

Lane 1 - WT CPMV, Lane 2 - F192W

1 2 3 4

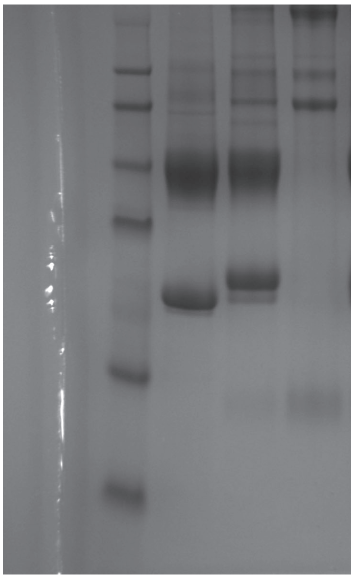

Lane 1 - Marker, Lane 2 - WT CPMV, Lane 3 - F192W infiltrated leaves, Lane 4 - F192W systemic infection

Gels from figure 5b

1 2 3

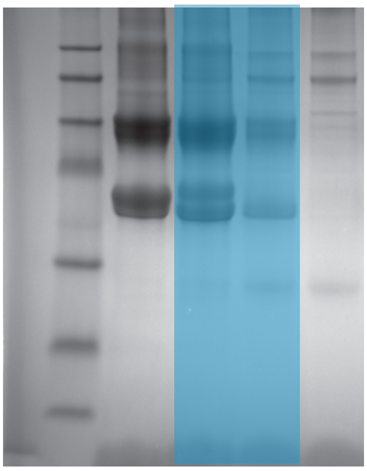

Lane 1 - Marker, Lane 2 - WT eVLP, Lane 3 - V109W

Gel from figure 5c

1 2 3 4 5

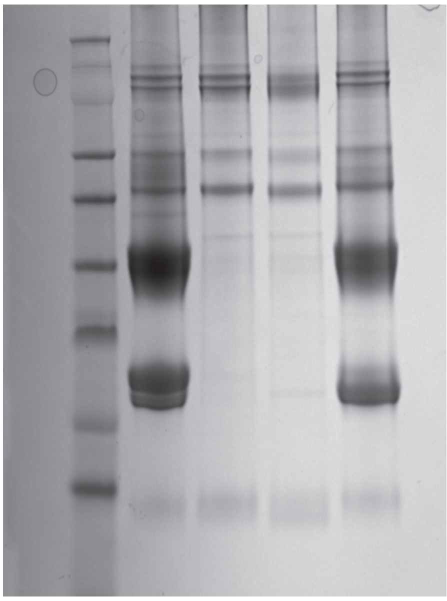

Lane 1 - Marker, Lane 2 - WT eVLP, Lane 3 - R193D, Lane 4 - E147R, Lane 5 - E147R/R193D

Supplementary Figure 2 - Gels from figure 5

Uncropped images of the lanes analysed to provide data for this manuscript in figure 5. Lanes not described are covered in a transparent blue box to avoid confusion.

Gels from figure 7a

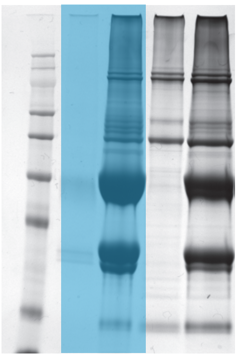

Lane 1 - Marker,
Lane 2 - R17E,
Lane 3 - WT eVLP

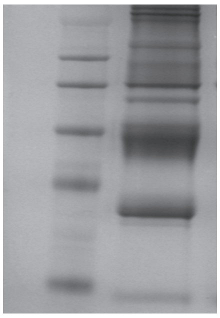

Lane 1 - Marker,
Lane 2 - R17D

Gels from figure 7b

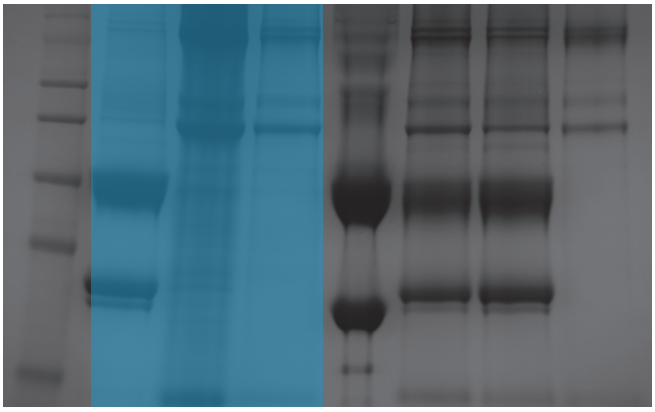

Lane 1 - Marker, Lane 2 - WT CPMV, Lane 3 - R17W, Lane 4 - R17G, Lane 5 - R17D

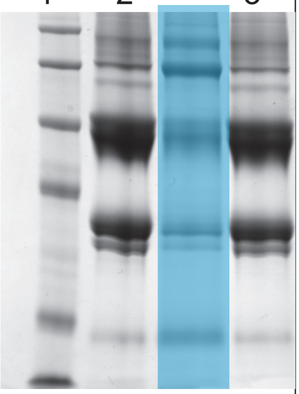

Lane 1 - Marker,
Lane 2 - WT CPMV,
Lane 3 - R17K

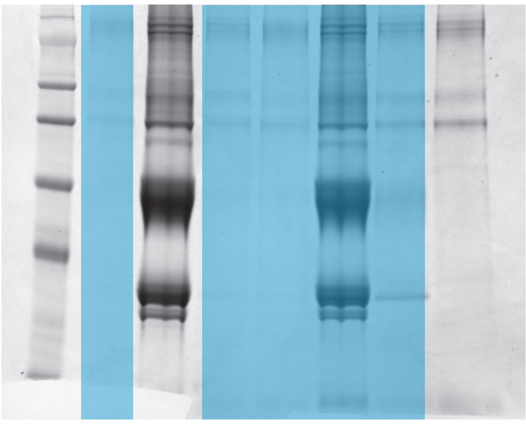

Lane 1 - Marker, Lane 2 - WT CPMV, Lane 3 - R17E

Gel from figure 7c

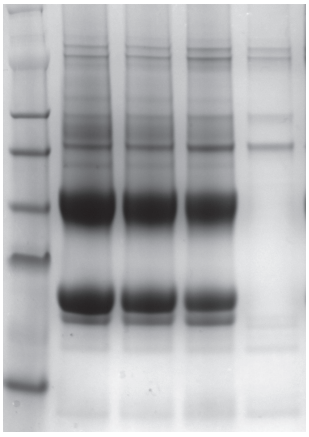

Lane 1 - Marker, Lane 2 - WT CPMV, Lane 3 - W190A, Lane4 - W190D, Lane 5 - W190F

Supplementary Figure 3 - Gels from figure 7
Uncropped images of the lanes analysed to provide data for this manuscript in figure 7. Lanes not described are covered with a transparent blue box to avoid confusion.

a

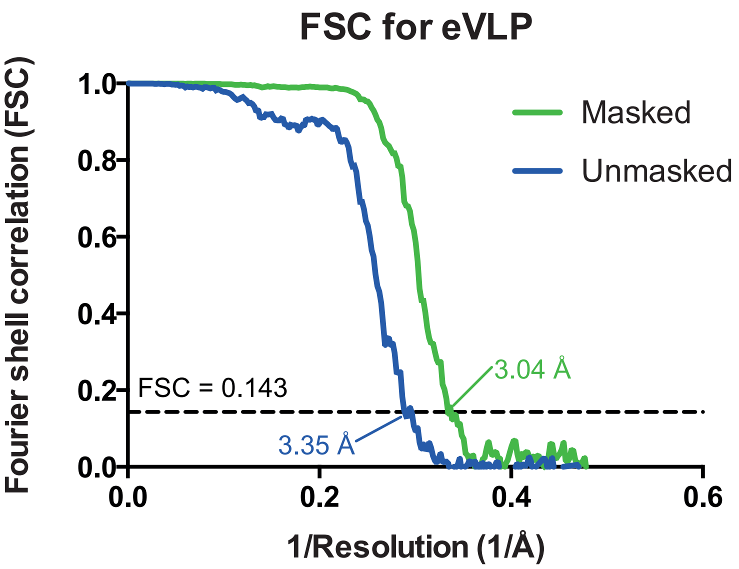

b

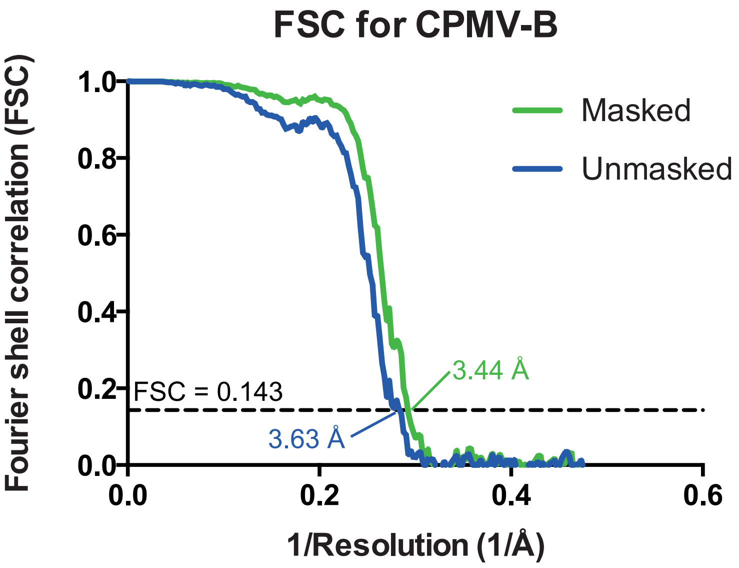

Supplementary Figure 4. Fourier shell correlation plots for eVLP and CPMV-B

Gold-standard Fourier shell correlation (FSC) plot for CPMV eVLP (a) and CPMV-B (b). The FSC plots were calculated for the final masked map (green) and the unmasked map (blue). Reported resolutions were based on FSC = 0.143 criteria.

SUPPLEMENTARY TABLES

Supplementary Table 1. C-terminal deletion mutants for eVLP CPMV

Deletion mutant	Number of C-terminal amino acids cleaved	Yield	Assembly
C-term Δ7	7	Wild-type (WT)	+++
C-term Δ11	11	WT	+++
C-term Δ14	14	Very low)	+
C-term Δ16	16	Very low	+
C-term Δ24	24	Very low	+

Supplementary Table 2. Effect of mutations on eVLPs assembly and yield after transient expression.

**Mutation**	**Capsid assembly**	**Yield**
Wild-type (WT)	Yes	as WT
F192W (S)	Yes	WT
F192H (S)	Yes	WT
F192Y (S)	Yes	WT
F194W (S)	Yes	WT
V109W (S)	No	No
V109D (S)	No	No
R193G (S)	No	No
R193D (S)	No	No
E147R (S)	No	No
E147R/R193D (S)	Yes	WT
R195G (S)	Yes	WT
R17D (L)	Yes	WT
R17E (L)	No	No

CPMV eVLP mutants were designed and transiently expressed (as described in the methods). The ability of the eVLP to form particles and the yield produced were analysed by SDS-PAGE and negative stain electron microscopy (EM). Mutations of small (S) subunit and large (L) subunit are indicated in brackets.

Supplementary table 3. Effect of mutations on CPMV capsid assembly, RNA encapsidation and systemic transport during infection.

**Mutation**	**Capsid assembly**	**RNA packaging**	**Systemic transport**
F192W (S)	Yes	No	No
F192H (S)	Yes	Yes	Yes
F192Y (S)	Yes	Yes	Yes
F194W (S)	Yes	Yes	No
R17D (L)	Low	No	No
R17E (L)	No	No	No
R17W (L)	Yes	Yes	Yes
R17K (L)	Yes	Yes	Yes
R17G (L)	Yes	Yes	Yes
W190A (L)	Yes	Yes	Yes
W190D (L)	Yes	Yes	Yes
W190F (L)	No	No	No

WT CPMV genomic RNA was mutated and CPMV particles produced as described in the methods. The ability of the CPMV mutants to form particles, the yield produced and the ability to produce systemic infection were analysed by SDS-PAGE and negative stain EM. Mutations of small (S) subunit and (L) large subunit are indicated in brackets.

Supplementary Table 4. Primers used for site-directed mutagenesis

Primer name	Primer sequence (5' – 3')
R17K-F	CCTTTCTTTGGATGATACAGGCTCAGTTAAGGGTTCTTTGCTTGACACAAAATTCG
R17K-R	CGAATTTTGTGTCAAGCAAAGAACCCTTAACTGAGCTTGTATCATCCAAAGAAAGG
R17E-F	CTTCTTTGGATGATACAAGCTCAGTTGAGGGTTCTTTGCTTGACACAAAATTC
R17E-R	GAATTTTGTGTCAAGCAAAGAACCCTCAACTGAGCTTGTATCATCCAAAGAAAG
R17A-F	TTTGGATGATACAAGCTCAGTTGCTGGTTCTTTGCTTGACAC
R17A-R	GTGTCAAGCAAAGAACCAGCAACTGAGCTTGTATCATCCAAA
R193G-F	ACGGAAAACTCCACCGTTATTAAAGTTTGGGTTTCGGGATATT
R193G-R	AATATCCCGAAACCCAAACTTTAATAACGGTGGAGTTTCCGT
E147D-F	ATCAGACCACCTGGTATCTTGATTGTGTTGCTACC
E147D-R	GGTAGCAACACAATCAAGAATACCAGGTGGTTCTGAT
E147R-F	ATCAGACCACCTGGTATCTTGACTGTGTTGCTACC
E147R-R	GGTAGCAACACAGTCAAGATACCAGGTGGTCTGA
F192W-F	GTCAACGGAAACTCCACCGTTATTAAAGTGGAGGTTTCGGGATATT
F192W-R	AATATCCCGAAACCTCCACTTTAATAACGGTGGAGTTTCCGTTGAC
F192Y-F	GGAAACTCCACCGTTATTAAAGTATAGGTTTCGGGATAT

	TGA
F192Y-R	TCAATATCCCGAAACCTATACTTTAATAACGGTGGAGTTTCC
F192H-F	CAACGGAAACTCCACCGTTATTAAAGCATAGGTTTCGGGATATTGAAC
F192-R	GTTCAATATCCCGAAACCTATGCTTTAATAACGGTGGAGTTTCCGTTG
F194W-F	GGAAACTCCACCGTTATTAAAGTTTAGGTGGCGGGATATTGAACGC
F194W-R	GCGTTCAATATCCCGCCACCTAAACTTTAATAACGGTGGAGTTTCC
R195G-F	TCCACCGTTATTAAAGTTTAGGTTTGGGGATATTGAACGCT
R195G-R	AGCGTTCAATATCCCCAAACCTAAACTTTAATAACGGTGGA
R193D-F	CTGTCAACGGAAACTCCACCGTTATTAAAGTTTGATTTTCGGGATATTGAACGC
R193D-R	GCGTTCAATATCCCGAAAATCAAACTTTAATAACGGTGGAGTTTCCGTTGACAG
V109W-F	GTTATGATGCGCGGACATTTTGGATCTCACAACCTGGTTCT
V109W-R	AGAACCAGGTTGTGAGATCCAAAATGTCCCGCGCATCATAAC
V109D-F	TATGATGCGCGGACATTTGATATCTCACAACCTGGTTCTG
V109D-R	CAGAACCAGGTTGTGAGATACAAATGTCCGCGCATCATA
V42D-F	CAACGGCAAAATAACTCCTGATGGTGATGACAATTGGAATA

V42D-R	TATTCCAATTGTCATCACCATCAGGAGTTATTTTGCCGTTG
V42W-F	GACTTAATCAACGGCAAAATAACTCCCCGGGGTGATGACAATTGGAATA
V42W-R	GTGCGTATTCCAATTGTCATCACCCCAAGGAGTTATTTTGCCGTTGATTAAGTC
W190A-F	CACCATTTACCACTTGGCTGATTGTCAGAATGCTTTACCCCTTAATCGTTG
W190A-R	CAACGATTAAGGGGTAAAGCATTCTGACAATCAGCCAAGTGGTAAATGGTG
W190F-F	CCACTTGGCTGATTGTCAGAATTTTTTACCCCTTAATCGTTGGA
W190F-R	TCCAACGATTAAGGGGTAAAAATTCTGACAATCAGCCAAGTGG

F – forward primer

R - reverse primer

Supplementary Table 5. Primers used for making deletion mutants in the C-terminus of the small coat protein

Primer name	Primer sequence (5' – 3')
DM-F	TACAATC<u>TCCGGA</u>AGATTTTAATCTTGG
DM24-R	GAC<u>AGGCCT</u>CTATAACGGTGGAGTTTC
DM17-R	TAC<u>AGGCCT</u>CTAAATTATCCCGAAACCT
DM7-R	TAC<u>AGGCCT</u>CTAAACCTAAACACTACG
DM11-R	TAC<u>AGGCCT</u>CTAACGCTTGGAGCGTTCAATATCCCG

Underline indicates position of restriction site, either BspEI or StuI.
